# Boron Nitride Nanotube Addition Enhances the Crystallinity and Cytocompatibility of PVDF-TrFE

**DOI:** 10.3389/fchem.2019.00364

**Published:** 2019-05-21

**Authors:** Anup Poudel, Marc A. Fernandez, Syed A. M. Tofail, Manus J. P. Biggs

**Affiliations:** ^1^CURAM, SFI Centre for Research in Medical Devices, National University of Ireland Galway, Galway, Ireland; ^2^Department of Physics, and Bernal Institute, University of Limerick, Limerick, Ireland

**Keywords:** Piezoelectric materials, tissue engineering, PVDF-TrFE/BNNT, MDSC, crystallinity

## Abstract

Analysis of the cellular response to piezoelectric materials has been driven by the discovery that many tissue components exhibit piezoelectric behavior *ex vivo*. In particular, polyvinylidene fluoride and the trifluoroethylene co-polymer (PVDF-TrFE) have been identified as promising piezo and ferroelectric materials with applications in energy harvesting and biosensor devices. Critically, the modulation of the structural and crystalline properties of PVDF-TrFE through annealing processes and the addition of particulate or fibrous fillers has been shown to modulate significantly the materials electromechanical properties. In this study, a PVDF-TrFE/boron-nitride nanotube composite was evaluated by modulated differential scanning calorimetry to assess the effects of boron nitride nanotube addition and thermal annealing on the composite structure and crystal behavior. An increased beta crystal formation [f(β) = 0.71] was observed following PVDF-TrFE annealing at the first crystallization temperature of 120°C. In addition, the inclusion of boron nitride nanotubes significantly increased the crystal formation behavior [f(β) = 0.76] and the mechanical properties of the material. Finally, it was observed that BNNT incorporation enhance the adherence and proliferation of human tenocyte cells *in vitro*.

## Background and Introduction

Piezoelectric polymeric materials have shown great promises in biomedical engineering, finding applications as pressure/tactile sensors for catheters (Sharma et al., 2012), energy harvesting devices for orthopedic implants (Safaei et al., 2018) and ultrasonic devices for piezoelectric ablation/surgery (Labanca et al., 2008). Recently, piezoelectric polymers and their composites have garnered special interest as active scaffolds for tissue engineering applications due to their facile processibility, flexibility, biocompatibility, and electromechanical properties, and have shown to promote cell functionality, proliferation and growth *in vitro* (Hitscherich et al., 2016; Wu et al., 2018).

The ferroelectric, piezoelectric and pyroelectric properties of these polymers, depends on the polymorphic crystalline phases present within their semi-crystalline polymer matrix (Boccaccio et al., 2002; Gregorio, 2006; Lutkenhaus et al., 2010), which can be modulated through various physicomechanical processing techniques (Karode et al., 2018). In particular, Poly(vinylidene fluoride) [PVDF], a semi-crystalline fluorinated polymer has been studied extensively as a ferroelectric material, demonstrating five different crystal phases (α-phase, β-phase, α]-phase γ-phase, ε-phase, and δ-phase) (Cui et al., 2015). Among these, β-phase rich PVDF formulations have received much attention due to their pyroelectric and piezoelectric properties (Wu et al., 2019). Although β-phase crystalinity in melt or drop-casted PVDF is relatively low, multiple studies have reported that post-processing approaches can increase the formation of the β-phase content in PVDF (Gregorio, 2006; Martins et al., 2014; Cui et al., 2015). In particular, the addition of fillers, through annealing with varying pressures and temperatures and controlled cooling rates, mechanical stretching, and electric poling have been reported to modulate PVDF β-phase content.

In addition, the copolymer Poly(vinylidene fluoride)- trifluoro ethylene (PVDF-TrFE) possesses an increased β-phase content relative to PVDF, without the need for further processing requirements (Martins et al., 2014). It has been reported however, that annealing of PVDF-TrFE between the Curie and melt temperatures, further increases the β-phase crystallinity, due to enhanced molecular movement with increased temperature (Zeng et al., 2009; Lau et al., 2013). The addition of micro and nanoscale fillers has also been shown to alter the piezoelectric, thermal and mechanical behavior of PVDF with the formation of additional phases between the polymer and the filler interface (Karode et al., 2018; Mayeen et al., 2018). However, the dynamics of polymorphism mechanisms in PVDF-TrFE and its composites with the addition of different nano/micro fillers are still not well-understood, restricting the use of piezoelectric composites as smart electromechanical materials.

A key tenet of tissue engineering is biomimetic design of materials capable of providing support for cell growth, proliferation, and differentiation. With respect to tendon tissue engineering, tenocyte-seeded scaffolds have been explored widely *in vitro* and *in vivo*; however, under conventional culture, tenocytes show rapid senescence and phenotypic drift. PVDF-TrFE has recently been explored as a biomaterial for orthopedic and tendon specific applications and has been shown to modulate differential cell function *in vitro* (Ribeiro et al., 2015; Hitscherich et al., 2016; Sobreiro-Almeida et al., 2017). Critically, cells have been shown to be highly sensitive to the presence of different PVDF crystal types, crystallinity, and surface properties (roughness, chemistry, and surface free energy) which necessitates tailored surface functionalization approaches (Ribeiro et al., 2012; Martins et al., 2014) (i.e., to enhance cell adhesion), resulting in modulated piezoelectric output, collectively affecting polymer cytocompatibility (Betz et al., 2003; Klee et al., 2003). Furthermore, PVDF and PVDF-TrFE demonstrate excellent resistance to chemical modification, leading to poor functionalization potential (Klee et al., 2003).

In this study, the percentage of β-phase crystallinity in PVDF-TrFE and a PVDF-TrFE/bornon nitride nanotube (BNNT) nanocomposite is evaluated under different annealing temperatures using Fourier-transform infrared spectroscopy (FTIR) and differential scanning calorimetry (DSC). In addition, the molecular movement and crystal formation process of ferroelectric PVDF-TrFE and PVDF-TrFE/BNNT composites, was assessed via modulated differential scanning calorimetry (MDSC) to evaluate the relaxation phenomena of these materials. Finally, in order to assess PVDF-TrFE/BNNT composites, as tendon specific biomaterials, the mechanical (tensile analysis) and cytocompatibility (*in vitro* studies with primary human tenocytes) was assessed in poly acrylic acid functionalized materials.

## Materials and Methods

### PVDF-TrFE Film Preparation

The PVDF-TrFE (70-30 mol %, MW = 300 kDa) copolymer was purchased from Solvay in powder form. Boron Nitride Nanotubes (BNNT) were obtained from BNNT Ltd. PVDF-TrFE was dissolved in a 1:1 ratio DMF/acetone mixture with a solute ratio of 5:1, and cast onto a glass plate to form films of 20–30 um thickness. BNNTs were subjected to ultrasonication for 4 h in a 1:1 ratio DMF/acetone mixture before the addition of PVDF-TrFE. Solution of PVDF-TrFE were subjected to varying drying temperatures as described below. After solvent evaporation, the films were further subjected to a subsequent 12 h of further annealing at the drying temperature.

### Differential Scanning Calorimetry (DSC) and Fourier-transform Infrared Spectroscopy (FTIR)

DSC experiments were carried out on all samples using a TA instrument 2000. Samples (5–8 mg) encapsulated in Tzeropans were quenched at a rate of 2°C/min to −75°C and kept under isothermal conditions for 3 min followed by heating at 10°C/min to 160°C follow by cooling at 10°C/min.

β-phase analysis was conducted with Attenuated Total Reflectance- Fourier Transfer Infrared Spectroscopy (ATR-FTIR). Infrared spectra of all materials and nanocomposites were obtained with a VARIAN 600 ATR-FTIR at room temperature. All data were collected between 600 cm^−1^ and 4000 cm^−1^ with an average of 32 individual scans. Using the absorption band of α and β phases at 532 cm^−1^ and 846 cm^−1^, the fraction of β-phase [F(β)] was calculated using the following equation

(1) $$\begin{array}{r} F\left(\beta \right)=\frac{X_{\beta }}{X_{\alpha }+X_{\beta }}=\frac{A_{\beta }}{{1.26A}_{\alpha }+A_{\beta }} \end{array}$$

where X_α_ and X_β_ are the crystalline mass fractions of the α and β phases and A_α_ and A_β_ correspond to absorption bands at 532 cm^−1^ and 846 cm^−1^, respectively. It was assumed that absorption bands follow the Beer-Lambert law with an absorption coefficient of K_α_ = 6.1 × 104 cm^2^/mol and K_β_ = 7.7 × 104 cm^2^/mol.

### Modulated Differential Scanning Calorimetry (MDSC)

MDSC experiments were carried out using a TA instrument 2000. An oscillation time period of 60 s and an amplitude of ±0.47°C was used during the heating and cooling cycles. Samples were purged with liquid nitrogen at 50 ml/min of cooling. Eight milligram of each sample were encapsulated and sealed in Tzero pans. For all experiments, samples were isothermally maintained for 3 min at the beginning and end of the heating and cooling runs and subjected to a sample quenching process from room temperature to −75°C. Samples were kept under isothermal conditions for 3 min followed by heating at 3°C/min. A similar method was previously implemented for microphase and molecular movement studies using a different copolymer (Karode et al., 2017; Poudel et al., 2017).

### Tensile Analysis

Tensile analysis of thin films was performed using a Zwick/Roell Z010 with a 1 kN load cell, a crosshead speed of 500 mm/min and a maximum extension of 500%. Thin films were prepared according to ASTM D 882. Results were presented as average values of *n* = 5 replicate experiments with standard deviation.

### Electrical Poling and Quasi-Static Measurement of Piezoelectric Coefficient

Electrical poling was carried out using direct current (dc) voltage. Thin films were clamped between two steel electrodes and subjected to an electric field of 100 V/um which was applied at 80°C for 10 min. The piezoelectric d33 coefficient of the thin films was measured by sandwiching the films between the electrodes which applied a quasi-static oscillatory force of 250 mN at a frequency of 111 Hz using a commercial piezometer (PM300, Piezotest, UK).

### Cytocompatibility Studies

#### Fibronectin Functionalization

Pristine PVDF-TrFE and PVDF-TrFE/BNNT substrates were oxygen plasma treated for 45 s at 30 W and with an oxygen flow rate of 30 ml/min to form surface hydroperoxides (Völcker et al., 2001). Subsequently, hydroperoxides decomposed due to thermal gradients to produced secondary radicals to initiate the polymerization of acrylic acid (AAc). After oxygen plasma polymerization, treated surfaces were immersed in a 20% v/v AAc aqueous solution previously purged with nitrogen at 90°C using a reflux system to avoid changes in concentration. Next, the pAAc functionalized surfaces were rinsed with distilled H20 for 18 h to remove unreacted monomers. Finally, caboxyl groups were activated with 0.1 M EDC and 0.1 M NHS (1:1) for 1 h. Immediately after films were immersed in a fibronectin solution (Sigma-aldrich, F1141-1MG) (10 ug/ml) for 2 h at room temperature.

#### Tenocyte Cell Culture

Human tendon derived cells were harvested from patellar tendon during tendon grafting operations after obtaining written informed consent. From these tissue specimens human primary tenocytes were isolated and cultured. All cells were maintained in Dulbecco's Modified Eagle's Medium (DMEM/F-12 with Glutamax, Gibco-BRL) supplemented with penicillin (100 U ml−1), streptomycine (10 μg ml^−1^) (both Sigma-Aldrich) and 10% fetal calf serum (Gibco-BRL). Cells were detached by incubation with 0.05% trypsin for 5 min at 37°C. They were identified as tenocytes through their characteristic growth pattern and by detection of scleraxis (SCX) and tenomodulin (TNMD) expression. The studies were performed with cells at passage 2–3 and a total of 3 donors were used for all assays.

#### Live/Dead Assay

Viable cells were seeded at a density of 5000 per well in 96-well (*n* = 5) plates for quantitative analysis and in 4-well glass bottom chamber slide for fluorescent microscopy. Tenocytes were cultured for 1, 3, or 7 days. Untreated live and dead cells were used as controls for quantitative analysis. A Live/Dead Assay (Life Technologies) was used to visualize viable and necrotic cells. After 1, 5, and 10 days, samples were washed with PBS and stained with calcein and ethidium bromide from the kit as recommended by manufacturer. The cells were incubated with the stock solution for 25 min and protected from light. The well plates were immediately analyzed with a Varioskan Flash plate reader. Samples were subsequently imaged on an Olympus IX81 inverted fluorescent microscopy with 20 × objective.

#### Cell Proliferation Assay

For cell proliferation analysis, tenocytes were seeded onto all experimental and control films in a 6-well plate at 4.5 × 10^4^ cells/film (*n* = 3). Cell metabolic activity and proliferation were assessed by Alamar Blue assay (Sigma-Aldrich). The cells were incubated in medium supplemented with 10% (v/v) Alamar Blue dye for 4 h and the absorbance at 570 and 590 nm measured in a 96-well plate using a Varioskan Flash Plate reader. Non-seeded biomaterial in the same medium was used as a negative control.

#### Statistical Analysis

Data was analyzed using Minitab 8 and was presented as mean ± SD from three or more separate experiments, as indicated. A Student's *T*-test (for single comparison) or one-way ANOVA (for multigroup comparisons). Tukey *post hoc* analysis was used to identify statistical significance.

## Results and Discussions

PVDF-TrFE and PVDF-TrFE/BNNT thin films were formulated via a drop-casting approach in a co-solvent of Acetone/DMF. It has been previously observed that although DMF is a better solvent for PVDF, acetone and DMF mixtures modulate the porosity and the dielectric breakdown strength of PVDF films (Chen et al., 2017) through control of solvent evaporation (California et al., 2011). In addition, solvent-cast PVDF-TrFE films formed through the evaporation of DMF at room temperature are associated with large diameter pores (μm sized) (Nunes-Pereira et al., 2015; Bodkhe et al., 2017; Ribeiro et al., 2018) which decreased with the use of a co-solvent of DMF and acetone without major alteration to the materials piezoelectric behavior (Chen et al., 2017). Furthermore, the addition of acetone to DMF favors low evaporation temperature with promotion of beta phase formation (Cui et al., 2015).

**Figure 1:**
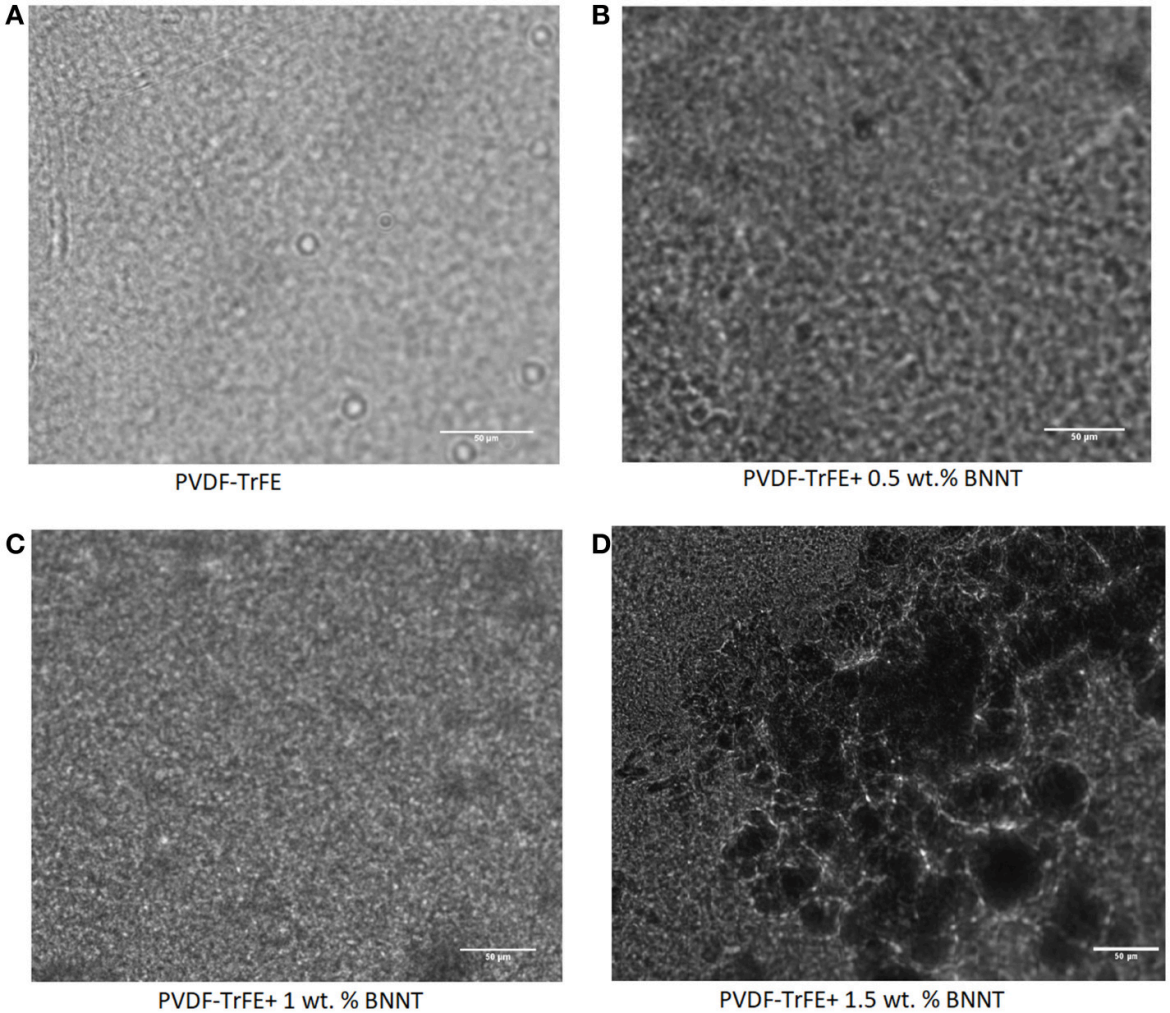
Digital microscopy images of **(A)** PVDF-TrFE **(B)** PVDF-TrFE+1% BNNT, **(C)** PVDF-TrFE+1% BNNT and **(D)** PVDF-TrFE +1.5% BNNT.

Digital brightfield microscopy of solvent-cast 0.5 wt.% and 1 wt.% BNNT nanocomposite films indicated BNNT dispersion without aggregation. Conversely, solvent-cast 1.5% w/v BNNT nanocomposite films were observed to contain BNNT agglomerations as (Figures 1A–D). However, use of further dispersal techniques as described previously could be used for better dispersion of BNNT at higher percentage in the polymer matrix and will be investigated in a follow-up study (Karode et al., 2018; Poudel et al., 2019). 1 wt.% BNNT nanocomposite films were subsequently selected for the focus of this study as the alteration of crystalline behavior of polymers upon the addition of a nanoscale filler is more pronounced when the fillers wt.% is sufficiently lower than the threshold concentration at which agglomeration occurs (Karode et al., 2018).

**Figure 2:**
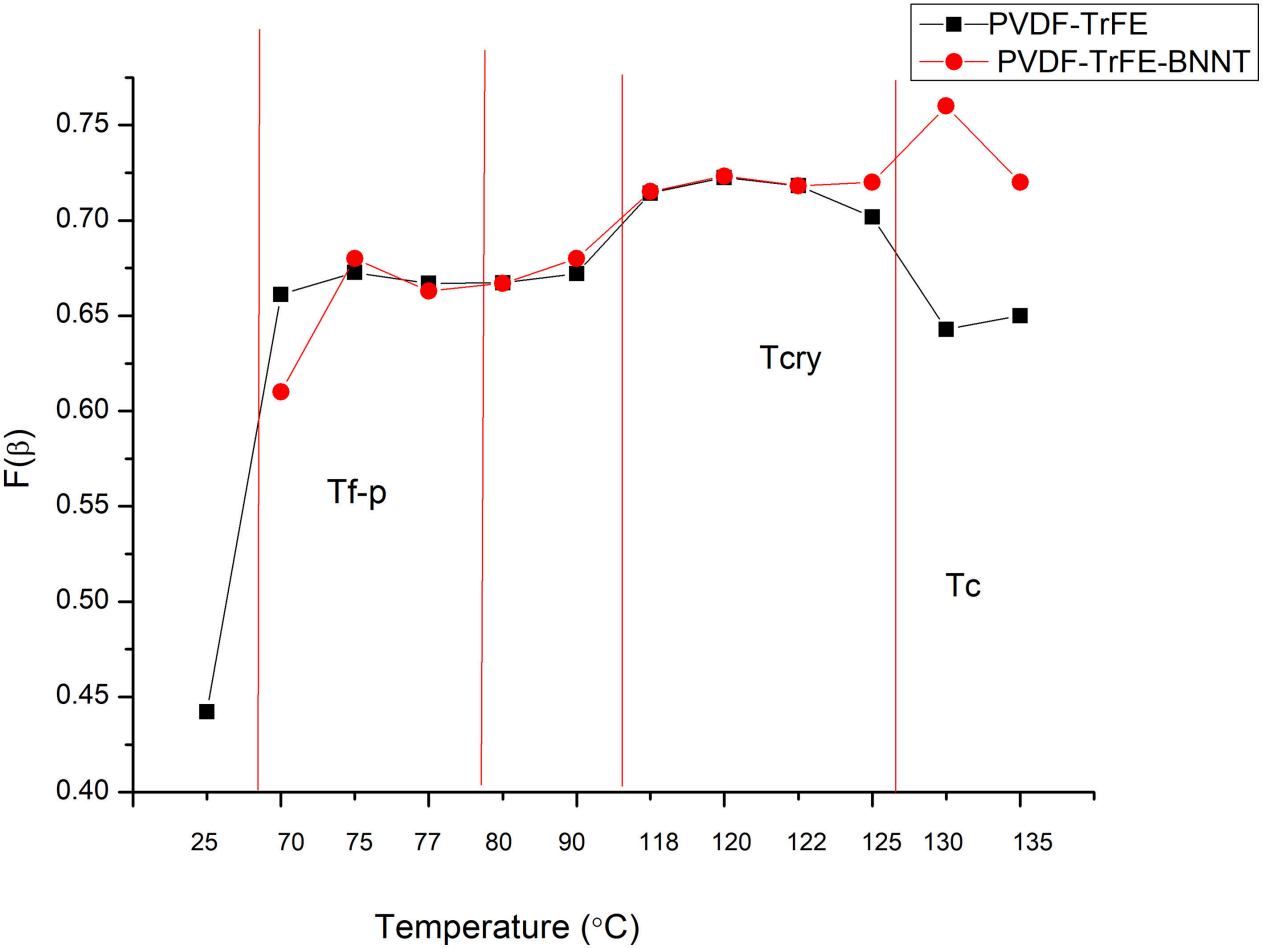
FTIR analysis of the β-phase fraction in pristine PVDF-TRFE and PVDF-TRFE/BNNT nanocomposites annealed under different temperature conditions for 12 h.

As the β-phase content of PVDF-TrFE and PVDF-TrFE composites has a direct impact on the piezoelectric properties of a polymer, the β-phase fraction was assessed using FTIR in samples subjected to a 12 h annealing process with temperatures ranging from 25 to 130°C. It was observed that the annealing temperature had a direct impact on the β-phase formation in PVDF-TrFE films, with the highest β-phase content in pristine PVDF-TrFE observed in samples annealed at 120°C, just above the T_c_ Curie temperature. Similarly, PVDF-TrFE/BNNT nanocomposites annealed at 120°C possessed a beta fraction [F(β)] equal to 0.72, similar to that of PVDF-TrFE annealed at 120°C (Figure 2). However, annealing of PVDF-TrFE/BNNT at 130°C resulted in a higher F(β) value, which was further investigated using DSC and MDSC techniques.

Conversely, annealing above 135°C was excluded as PVDF-TrFE started to melt above this temperature (Figure 2). Here, the F(β) was observed to decrease as the annealing temperature approached the T_m_ and to decrease significantly when the samples were subjected to room-temperature drying and annealing conditions. It has been reported previously that β-phase crystallinity is substituted by a high α-phase content when PVDF is quenched from the melt or annealed at room temperature (Ramasundaram et al., 2008). Ramasundaram et al. also observed that the fraction of β-phase, F(β) in PVDF decrease as the annealing temperature increases above 120°C. This was not observed with PVDF-TrFE in this study, perhaps due to the rapid β-phase crystallization occurring in PVDF-TrFE (Lutkenhaus et al., 2010).

**Figure 3:**
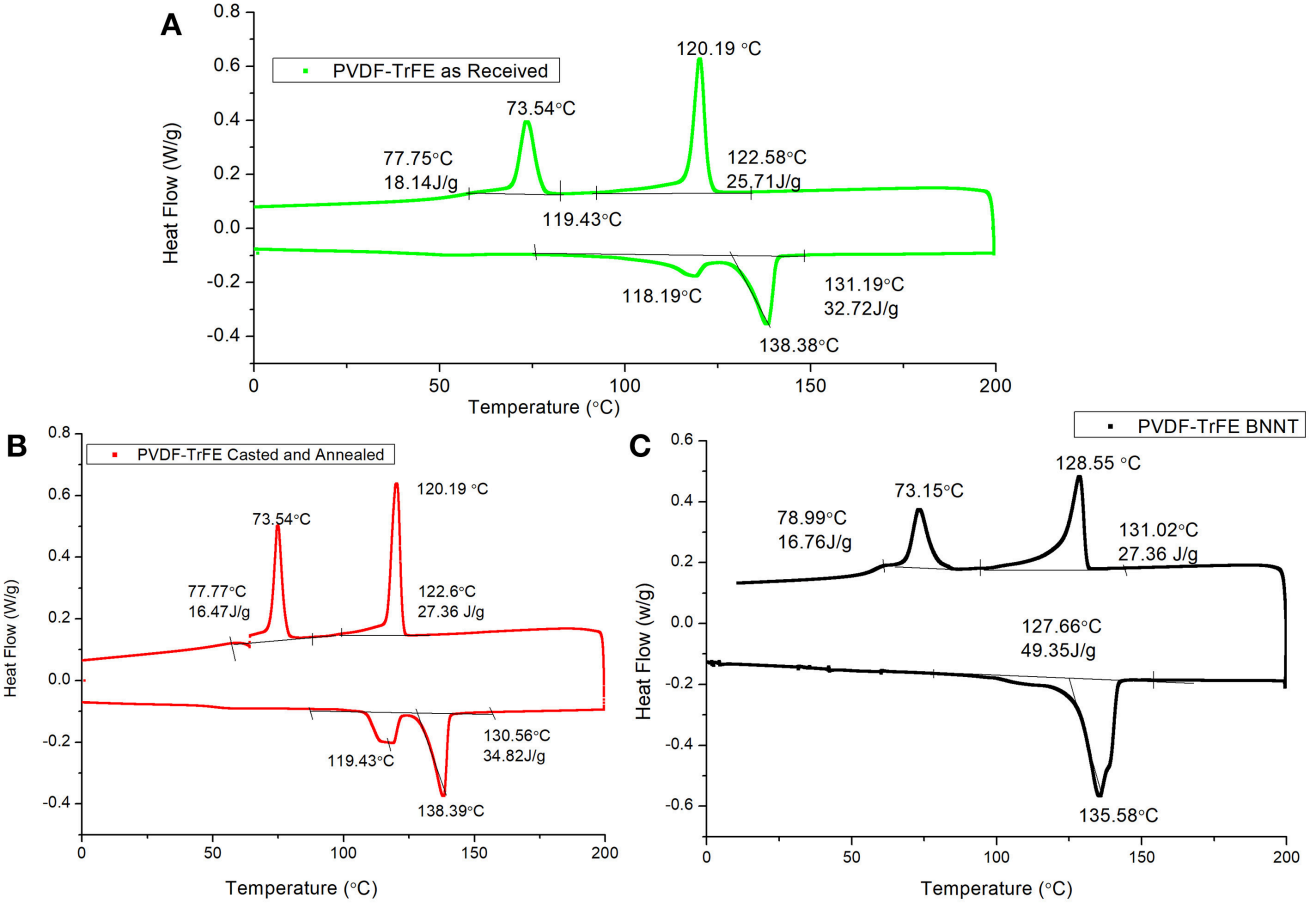
Representative DSC curves of **(A)** PVDF-TrFE as-received, **(B)** Solvent-cast PVDF-TrFE annealed at 120°C and **(C)** Solvent-cast PVDF-TrFE BNNT 1 wt.% nanocomposite annealed at 120°C.

Figures 3A–C shows representative DSC curves (heating and cooling of 5°C/min) of PVDF -TrFE (as-received), solvent-cast PVDF-TrFE annealed at 120°C, and solvent-cast PVDF-TrFE/BNNT 1 wt.% annealed at 120°C. During the heating and cooling cycles two clear peaks were observed for PVDF-TrFE samples representing crystal formation during the heating and cooling processes (Figure 3A). It has been hypothesized previously that the first endothermic peak represents the phase transformation (termed the ferroelectric Curie temperature), and is characteristic of piezoelectric polymers (Martins et al., 2014), while the second endothermic peak represents the polymer melting temperature (T_m_). After the copolymer was solvent cast and annealed at 120°C, the two melting peaks became narrow (unannealed PVDF-TrFE ranging from 118.1°C to 138.3°C and annealed PVDF-TrFE ranging from 119.4°C to 138.3°C), as a result of the increased content of all-trans chains in the copolymer (Figure 3B). The addition of BNNT resulted in an increase in the observed curie temperature of the polymer, yet no difference in the T_c_ and T_m_ temperature relative to pristine PVDF-TrFE. The T_c_ in PVDF-TrFE/BNNT 1wt.% nanocomposites occurred over a broad range which is thought to be due to the formation of a large number of crystals and overlapping thermal events (Figure 3C).

In addition, the observed enthalpy of the heat of melting and total heat flow (ΔH) was greater in PVDF-TrFE/BNNT 1wt.% nanocomposites, suggesting an enhanced crystallinity of the PVDF-TrFE matrix through the addition of BNNTs. During the cooling cycle, the first crystal formation occurred at ~120°C, although crystal formation was initiated at ~122°C. It has been hypothesized that the paraelectric to ferroelectric transition occurs at low temperatures in PVDF, which was identified here to occur at 73°C.

The low heat of enthalpy noted in as-received PVDF-TrFE samples relative to solvent-cast PVDF-TrFE films annealed at 120°C and solvent-cast PVDF-TrFE/BNNT 1 wt.% nanocomposites annealed at 120°C suggested a low crystallinity was present in PVDF-TrFE (as-received). The percentage of heat of enthalpy, which is directly corelated to percentage of crystallinity, of PVDF-TrFE was increased by 6.5% when annealed at 120°C and by 50% with the addition of BNNT 1 wt.% compared to PVDF-TrFE as-received. A significant difference in total crystallinity however was also noted between PVDF-TrFE annealed and PVDF-TrFE/BNNT, which both possessed a high crystallinity.

In order to assess a correlation between polymer crystallization and molecular moment phenomena in pristine PVDF-TrFE (as-received), solvent-cast PVDF-TrFE films annealed at 120°C and solvent-cast PVDF-TrFE/BNNT 1 wt.% nanocomposites annealed at 120°C, modulated differential scanning calorimetry (MDSC) was performed to resolve overlapping kinetic events into reversing and non-reversing DSC curves. This technique has been recently used to characterize complex block polymers, but has not been fully utilized to characterize the molecular movement and crystallization behavior of PVDF-TrFE (Knopp et al., 2016; Karode et al., 2017).

**Figure 4:**
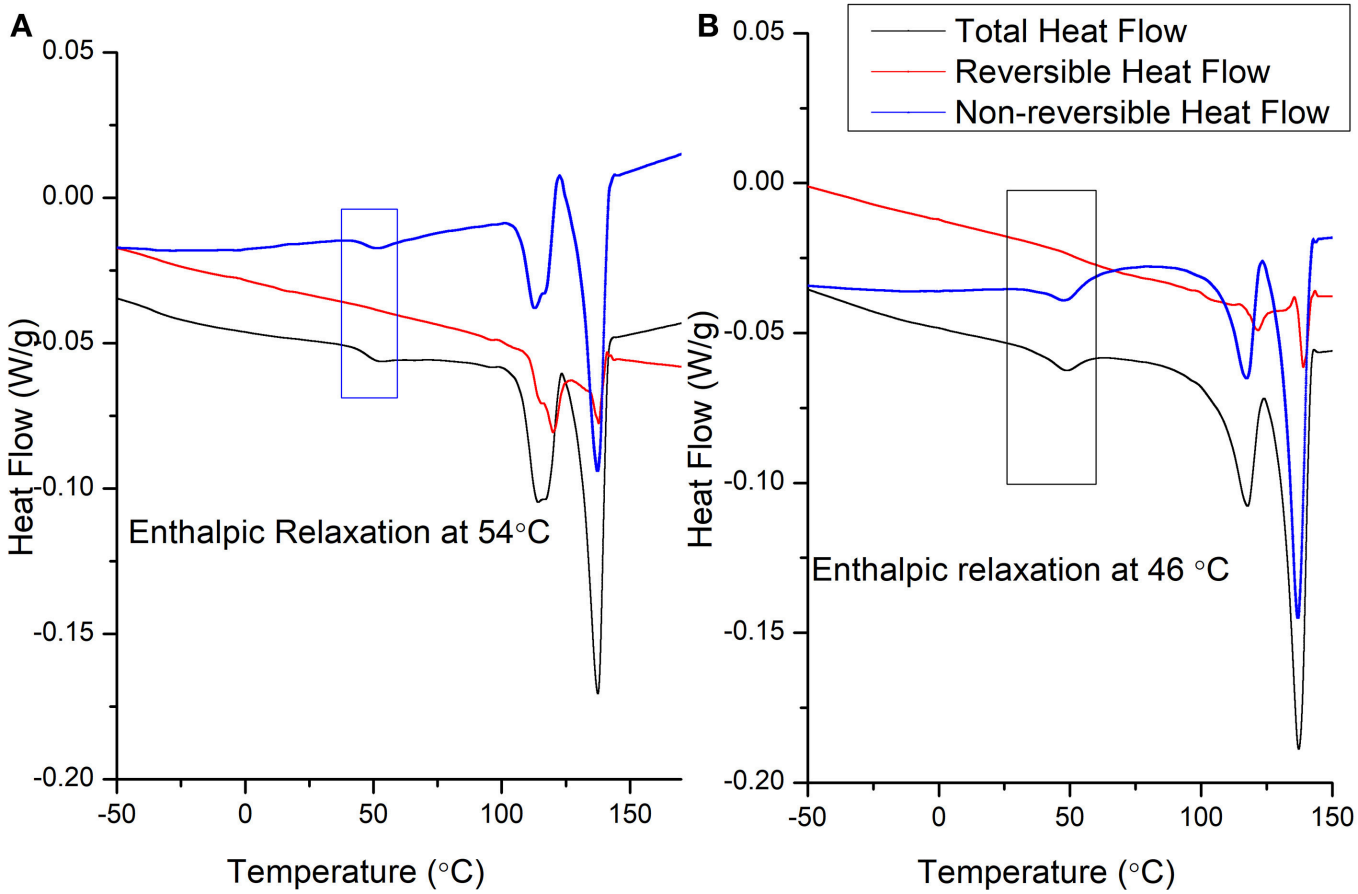
Representative MDSC heating cycle curves of **(A)** as-received PVDF-TrFE and **(B)** solvent-cast PVDF-TrFE films annealed at 120°C.

Figures 4A,B indicate the MDSC curves of as-received PVDF-TrFE and solvent-cast PVDF-TrFE films annealed at 120°C. The total heat flow is divided into two main events (i) a reversing curve, which directly measures the specific heat capacity, which in turn is a measure of the change in the molecular movement within a polymer occurring in a time scale shorter than a modulation time period (Lutkenhaus et al., 2010; Karode et al., 2017). Similarly, (ii) the non-reversing curve, indicates kinetic phenomena which occur with a time duration greater than the modulation time period. The first transition, an enthalpic relaxation was observed in as-received PVDF-TrFE at ~54°C yet was shifted to 47°C in solvent-cast PVDF-TrFE films annealed at 120°C. Previous studies suggest that this shift in the enthalpic relaxation temperature is due to the phase segregation within a copolymer which occur during the thermal annealing process. This type of thermal relaxation observed in copolymers is responsible for the initiation of the molecular movement which occur at the interface of two thermally incompatible phase domains, i.e., crystalline-amorphous domains or different crystalline-crystalline domains. The second endothermic peaks observed at ~125°C and 138°C in as-received PVDF-TrFE and solvent-cast PVDF-TrFE films annealed at 120°C respectively represent kinetic phenomena, as demonstrated by the non-reversing curve. The initial endothermic peak, observed at ~125°C was followed by an exothermic peak at ~129°C (cold crystallization) in the total and non-reversible curve of both annealed and non-annealed PVDF-TrFE indicates fusion of the metastable phase of the copolymer into more stable crystal forms. Due to the kinetic phenomena observed prominently in the non-reversible heat flow curve relative to the reversible heat flow curve. The final endothermic peak at ~138°C is assigned to the fusion of stable crystals and is clearly observed in the reversable as well as non-reversible heat flow curves in both as-received and annealed PVDF-TrFE formulations, suggesting the final melting point of all formed crystals.

**Figure 5:**
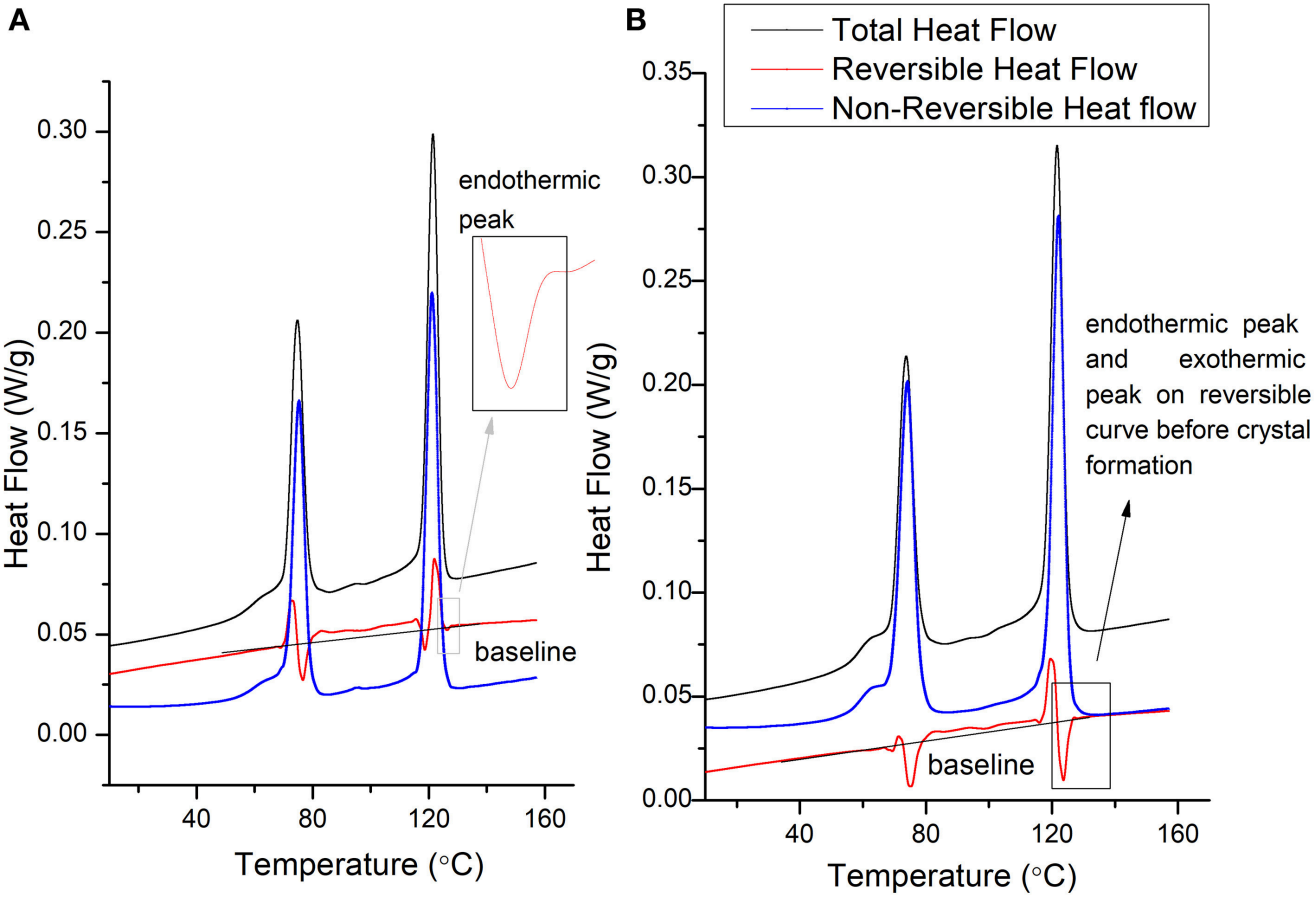
Representative MDSC cooling cycle curves of **(A)** as-received PVDF-TrFE and **(B)** solvent-cast PVDF-TrFE films annealed at 120°C.

The MDSC cooling cycle for as-received PVDF-TrFE (Figure 5A) and solvent-cast PVDF-TrFE films annealed at 120°C (Figure 5B) suggests that the crystallization phenomena represent a kinetic process, however, a change in the molecular movements (endothermic and exothermic phenomena in the reversible curve) as a function of specific heat capacity was observed during crystallization. The initiation of molecular movement in both solvent casted and as-received PVDF-TrFE (denoted by endothermic followed by exothermic phenomena) occurred just above the Curie temperature (the temperature at which a significant degree of crystals were formed in this copolymer), during the crystal formation process. The initial endothermic peak during the cooling cycle suggested a significant molecular movement before the initiation of crystal formation. This phenomenon was followed by a restriction of molecular movement due to the initiation of crystal formation and was denoted by an exothermic peak in the reversible cooling cycle curve. Previous studies suggest that during annealing of the polymer, a high degree of molecular movement occurs, favoring high β-phase crystallinity in annealed PVDF-TrFE (Lau et al., 2013). As observed in the reversible cooling cycle curve, the restricted molecular movement in as-received PVDF-TrFE occurred over the range of the experimental temperatures as the curve remained above the baseline, resulting in heterogeneous β-phase crystal formation. Conversely, solvent-cast PVDF-TrFE films annealed at 120°C were associated with a more homogenous crystal formation, as molecular movements were more restricted relative to the non-annealed samples.

Annealing of the copolymers at any temperature between the Curie and melt temperatures however, may not have provided sufficient molecular restriction for a high degree of β-phase crystallinity due to (a) the formation of temperature mediated phase-separated domains and (b) crystal formation from melt may not always occur in between the Curie and melt temperature observed at 118°C and 138°C respectively.

**Figure 6:**
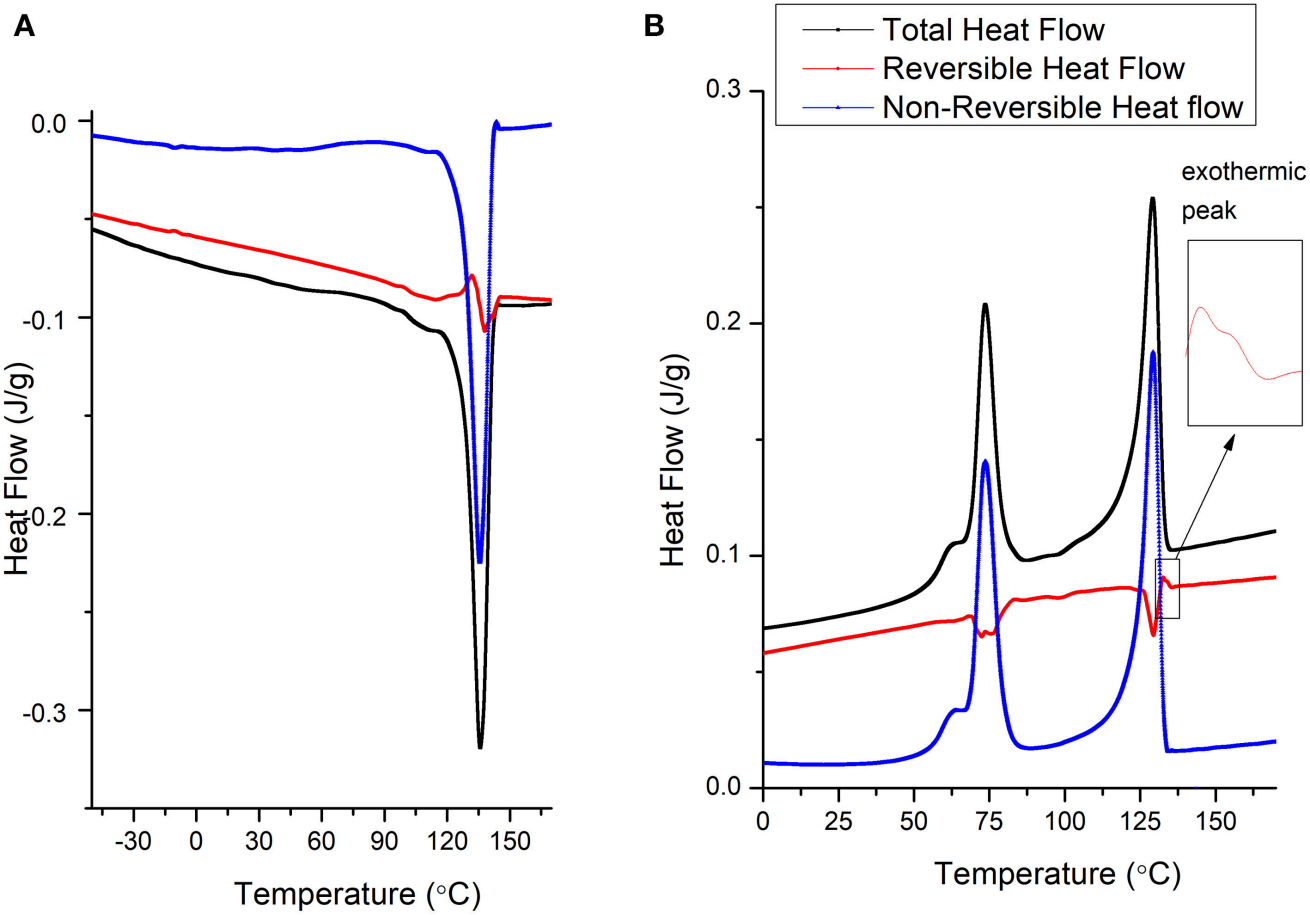
Representative MDSC curves of solvent-cast PVDF-TrFE BNNT 1 wt.% nanocomposites annealed at 120°C **(A)** heating cycle and **(B)** cooling cycle.

With the addition of 1 wt. % BNNT to the PVDF-TrFE matrix, no enthalpic relaxation was observed at low temperatures during the heating cycle, and the composite showed a single melting point as observed in the total heat flow curve (Figure 6A). This suggests a high interaction of BNNT with different PVDF-TrFE crystal domains, resulting in a slow relaxation of copolymer chains due to (a) high amorphous domain entanglement and (b) modulated inter-crystal crystallization. Confirmation of different crystal formation phenomena requires crystallization studies, which remains a major future study for this work. Moreover, due to the high interaction between polymer chains and the incorporated BNNTs, the modulation time used (60 s) was not long enough to observe the reversible process at a melting point in the reversible heat flow curve. However, the polymer chain movement occurred within 60 s for pristine PVDF-TrFE as observed in Figure 4.

**Figure 7:**
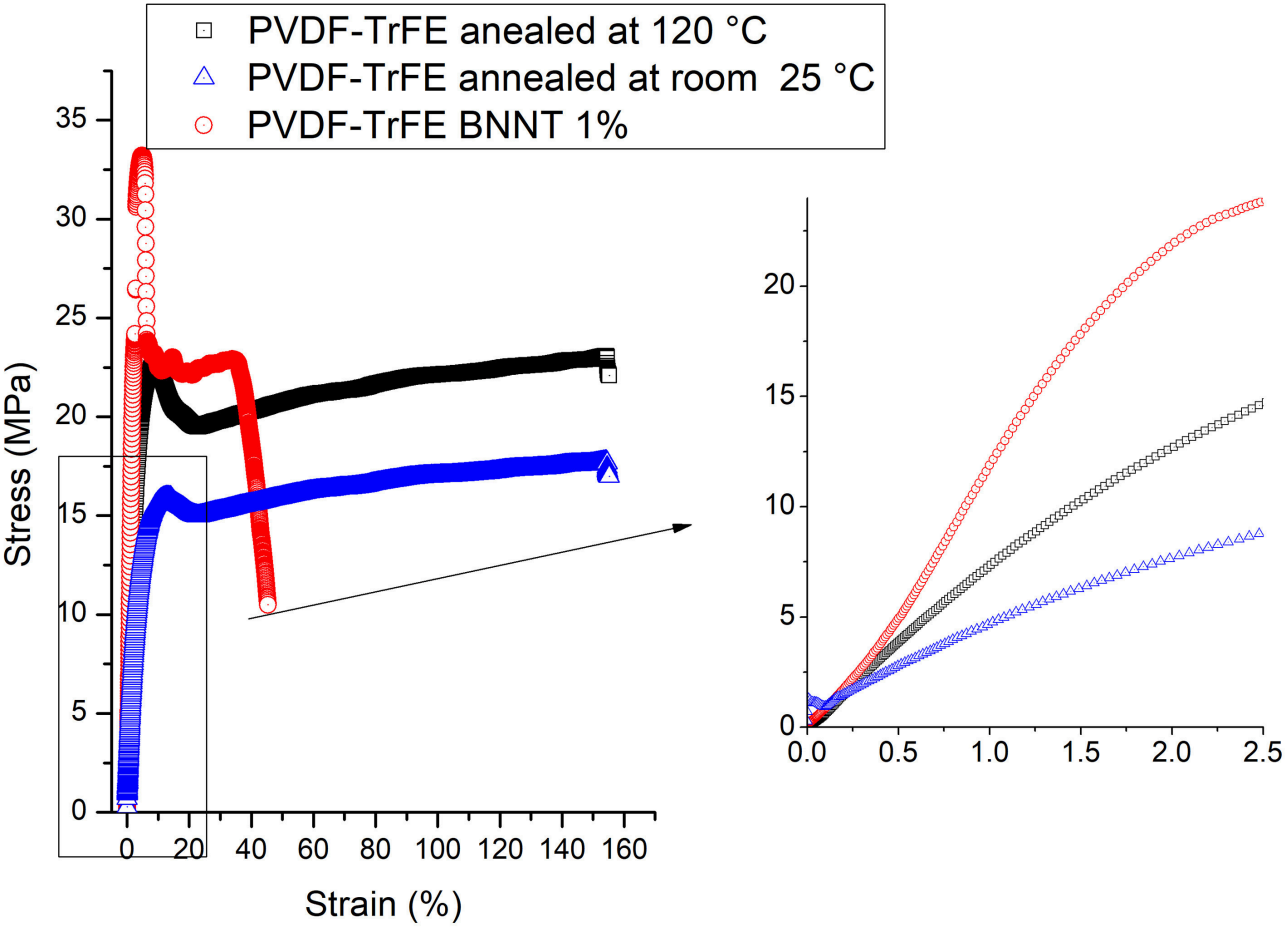
Tensile stress-strain analysis of solvent-cast PVDF-TrFE films annealed at room temperature and 120°C and solvent-cast PVDF-TrFE BNNT 1 wt.% nanocomposite films annealed at 120°C.

During the crystallization temperature of the cooling cycle (Figure 6B), solvent-cast PVDF-TrFE BNNT 1 wt.% nanocomposites annealed at 120°C demonstrated a low molecular movement in the reversing curve compared to both annealed and non-annealed PVDF-TrFE samples. However, crystals were formed mainly due to kinetics process, as observed in the non-reversible curves and minor molecular movement was observed during the crystal formation process at the crystallization temperature. Interestingly, initiation of restriction (a small exothermic peak on the reversing curve) occurred at 132°C in solvent-cast PVDF-TrFE BNNT 1 wt.% nanocomposites annealed at 120°C, a temperature 12°C higher than observed with pristine PVDF-TrFE formulations, suggesting the nucleation of crystals at higher temperatures through BNNT addition. This early restriction of molecular movement is also suggested by an observed increase in the viscoelastic properties of PVDF-TrFE BNNT 1 wt.% nanocomposites, resulting in an increased Young's modulus and modulus of resilience relative to both pristine PVDF-TrFE formulations (Figure 7; Table 1).

**Table 1:** Mechanical properties of solvent-cast PVDF-TrFE films annealed at room temperature and 120°C and solvent-cast PVDF-TrFE BNNT 1 wt.% nanocomposite films annealed at 120°C.

**Materials**	**Young's modulus** **(MPa)**	**Modulus of resilience** **(MPa)**	**Modulus of toughness (MPa)**
PVDF-TrFE annealed at RT	466.2 ± 38.8	51.77 ± 4.2	2517.01 ± 294.2
PVDF-TrFE annealed at 120°C	801.2 ± 32.8	65.72 ± 6.6	3307.06 ± 252.3
PVDF-TrFE and BNNT 1 wt % annealed at 120°C	1009.1 ± 56.2	112.26 ± 12.06	988.23 ± 102.3

In addition, the modulus of toughness and the percentage of elongation in pristine solvent-cast PVDF-TrFE annealed at room temperature decreased sharply with the addition of BNNT 1 wt. %, which was attributed to a high entanglement of BNNTs with the amorphous phase of PVDF-TrFE, leading to a low extension capability. Similarly, solvent-cast PVDF-TrFE annealed at 25°C was associated with a low Young's modulus and modulus of resilience. Failure did not occur at 150% strain in both pristine PVDF-TrFE samples, however, solvent-cast PVDF-TrFE BNNT 1 wt.% nanocomposites annealed at 120°C were observed to fail at 40% strain.

**Figure 8:**
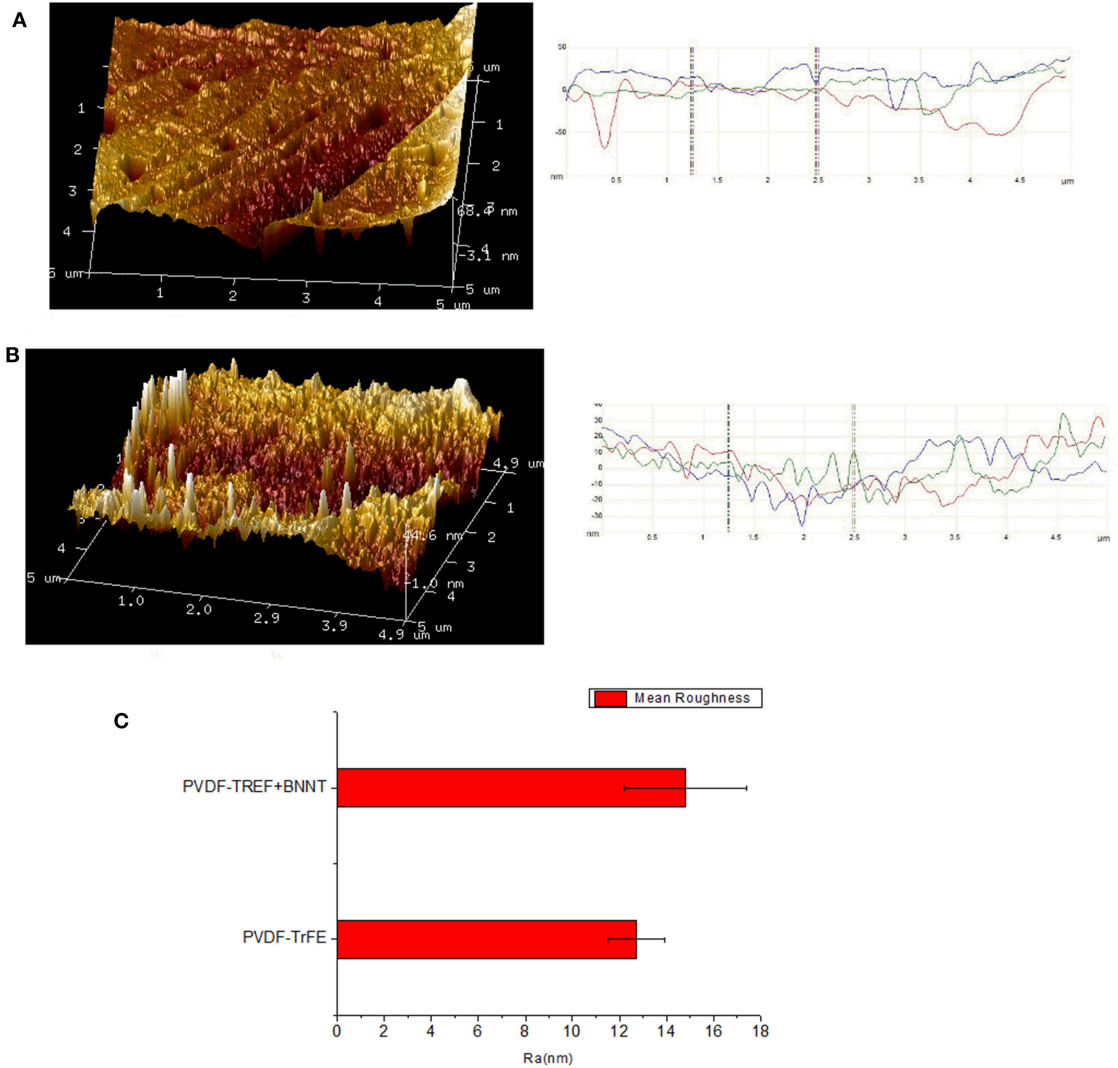
**(A)** AFM surface analysis of PVDF-TrFE and **(B)** PVDF-TrFE +1 wt.% BNNT. **(C)** Mean roughness (Ra) of PVDF-TrFE and PVDF-TrFE +1 wt.% BNNT.

AFM analysis of PVDF-TrFE and PVDF-TrFE BNNT 1 wt. % films was performed to assess surface topography and to measure the surface roughness (Figure 8). The mean surface roughness of solvent-cast PVDF-TrFE films annealed at 120°C and solvent-cast PVDF-TrFE BNNT 1 wt.% nanocomposite films annealed at 120°C was found to be 12.7 ± 1.2 nm and 14.8 ± 2.6 nm respectively. Although, the overall mean roughness between formulations was not statistically significant, the surface profile measured along the x, y plane by parallel line to x-axis at y equal to 1 (blue), 2 (red), and 3 (green) showed higher frequencies of roughness patterns in all BNNT composites compared to pristine PVDF-TrFE substrates. This analysis revealed that the addition of BNNT affected the surface morphology and frequency of surface roughness of PVDF-TrFE formulations, which could be directly corelated with the material crystallinity and crystal size as described by the MDSC process.

All unpolled sample types possessed a piezoelectric d33 coefficient of zero when assessed with quasi static direct piezoelectricity measurement. Both PVDF-TrFE and PVDF-TrFE-BNNT 1 wt. % BNNT nanocomposite annealed at 25°C, were associated with arcing during the poling operation, the occurrence of which was related to the presence of porosity in the polymer film. Conversely, solvent-cast PVDF-TrFE films annealed at 70°C demonstrated an improved ability to be poled to a maximum piezoelectric coefficient of 7 pC/N. These films however could not be poled with a field of 100 V/μm due to arcing. It is suggested that the evaporation rate of the co-polymer solvent was decreased at lower temperatures which caused defects in the film resulting in dielectric breakdown during poling. However, solvent-cast PVDF-TrFE films annealed at 120°C sustained a poling voltage of 100 V/μm, demonstrating improved dielectric breakdown strength due to lower porosity. These films showed a d33 coefficient of >11 pC/N. Similarly, solvent-cast PVDF-TrFE 1 wt. % BNNT nanocomposite films annealed at 120°C sustained higher poling voltages, relatively increased crystallinity and a piezoelectric d33 constant between 9 and 14 pC/N.

### *In-vitro* Analysis of Material Cytocompatibility

**Figure 9:**
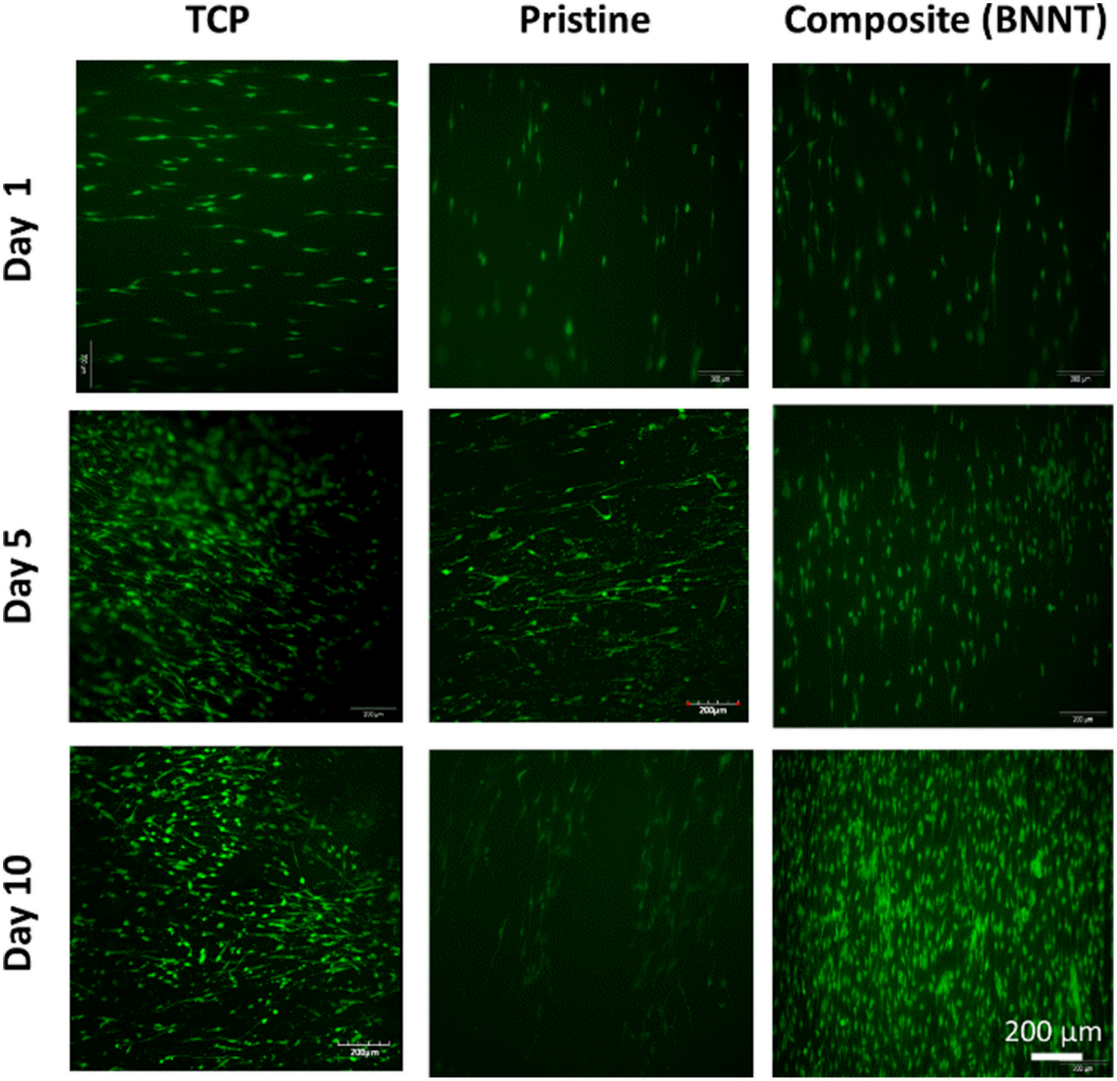
Live/dead assay of human tenocytes cultured for 1, 5 and 10 days *in vitro* on unpoled pristine solvent-cast PVDF-TrFE films annealed at 120°C and (B) solvent-cast PVDF-TrFE 1 wt. % BNNT nanocomposite films annealed at 120°C.

**Figure 10:**
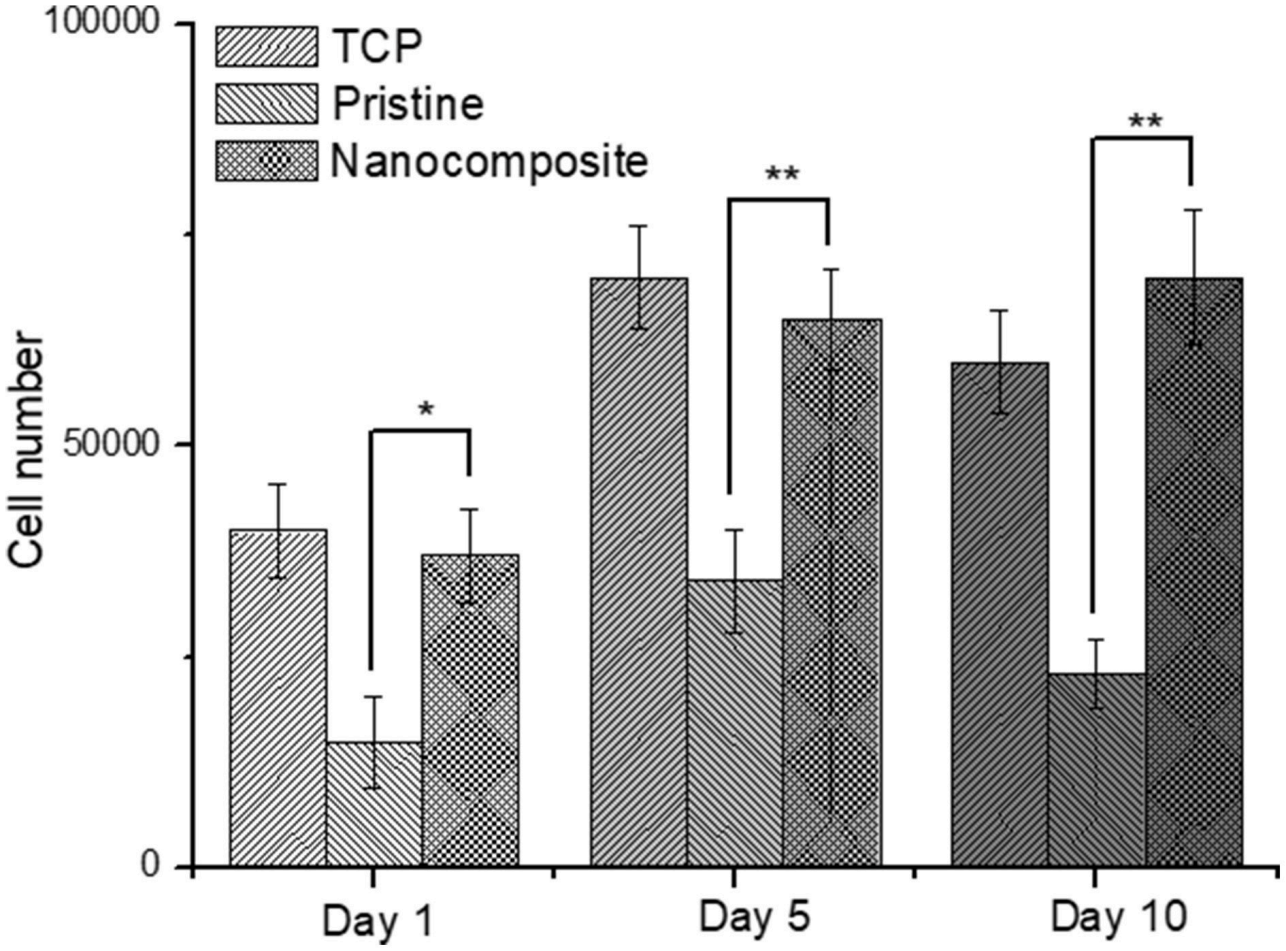
Total cell number of human tenocytes cells cultured on pristine annealed PVDF-TrFE and annealed PVDF-TrFE 1 wt. % BNNT nanocomposite films for 1, 5, and 10 days *in vitro*. Results are mean ± STD, * indicates a P-value of <0.05, ** indicates a P-value < 0.01 with respect to pristine non-poled samples.

In order to assess the ability of PVDF-TrFE materials to support tissue interfacing applications, annealed PVDF-TrFE and annealed PVDF-TrFE 1 wt. % BNNT nanocomposite films were progressed to cell viability studies for up to 10 days in culture, using tissue culture plastic (TCP) as a control substrate. It was observed that cell viability was maintained on all experimental and control substrates and significant cell death was not observed (Figure 9). However, cell proliferation assays confirmed that cells cultured on PVDF-TrFE/BNNT nanocomposites demonstrated enhanced proliferation for up to 10 days in culture relative to pristine annealed PVDF-TrFE films (Figure 10).

After 1 day in culture it was observed, that the incorporation of BNNT resulted in an increase in cell adhesion. In particular, a 2-fold increase in total cell number was observed on annealed PVDF-TrFE 1 wt. % BNNT nanocomposite films relative to cells cultured on pristine annealed PVDF-TrFE films. This trend was maintained at day 5 and was increased by day 10. This effect can be attributed to the observed nanotopography present on annealed PVDF-TrFE 1 wt. % BNNT nanocomposite films. Alternatively, it can be inferred that cell adhesion is enhanced by the electrically neutral surface of BNNTs that act as an anchoring site for cells (Giannini et al., 2018).

As previously demonstrated by Biggs et al. (2010, 2017), the cellular topographical sensing machinery is capable of detecting discrete differences in their extracellular environment down to the nanometer level (Fernandez-Yague et al., 2015). Similarly, Giannini et al. observed increased adhesion of fibroblasts cultured on surfaces characterized by protruding nanotubes. Furthermore, Zhang et al. (2015), demonstrated that cell Rho A–mediated cell proliferation increased with increasing surface roughness on surfaces produced by aggregation of nanotubes compared to flat surfaces. Finally, Genchi et al demonstrated that incorporation of BNNT in PVDF-TrFE can promote the differentiation of human osteoblasts *in vitro* (Genchi et al., 2018).

Although topographical factors contribute to the creation and maintenance of an optimal microenvironment for cell growth *in vitro*, a physical consideration of piezoelectric surfaces is the remnant polarization after poling which charges the materials surfaces. This electrical polarization can be either positive or negative and may affect cell adhesion or proliferation. In order to investigate the role of surface charge on tenocyte adhesion and proliferation, cells were cultured on polarized and unpolarized pristine annealed PVDF-TrFE and annealed PVDF-TrFE 1 wt. % BNNT nanocomposite films for 5 days.

**Figure 11:**
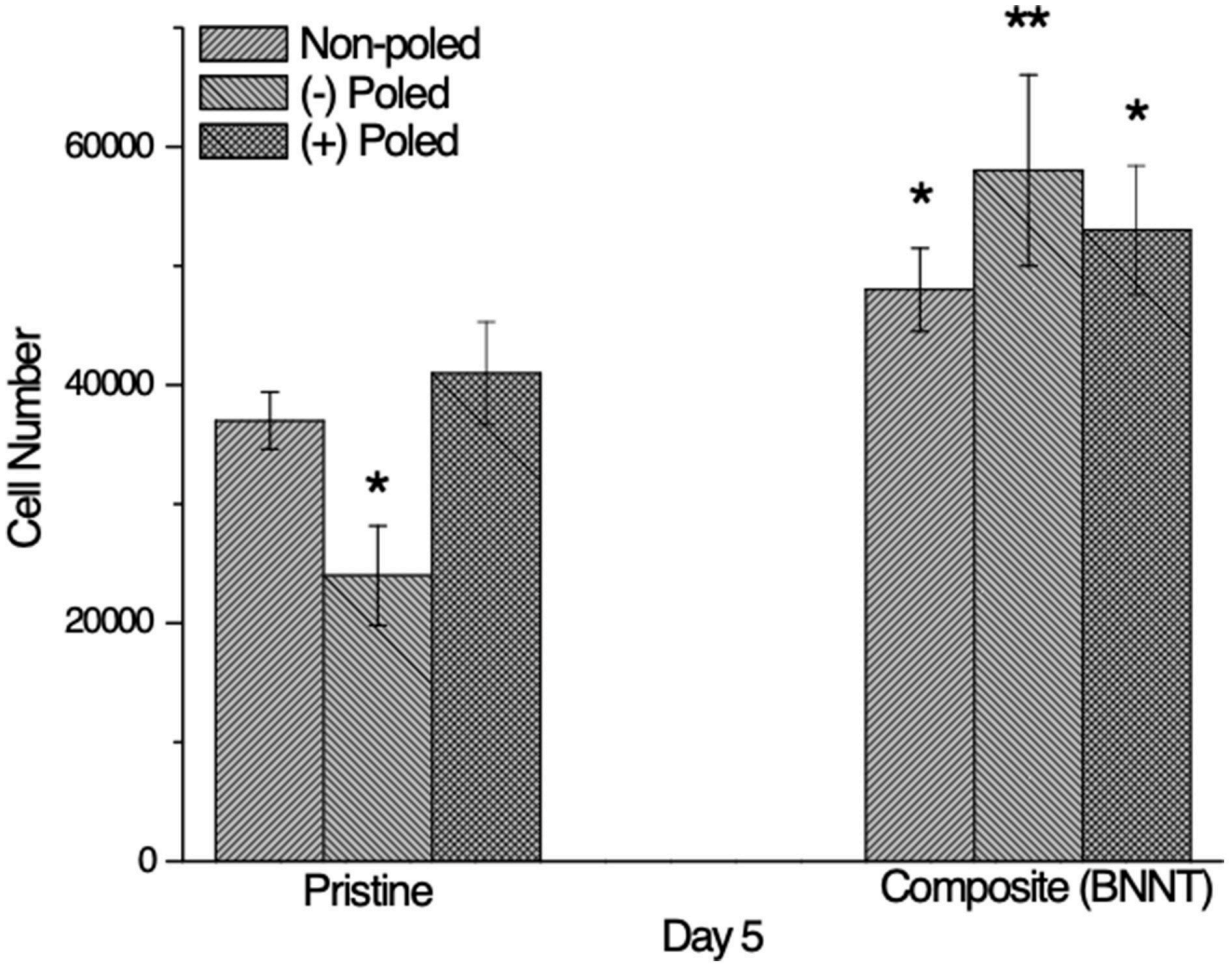
Total cell number of human tenocytes cells cultured on unpolarized and polarized pristine annealed PVDF-TrFE and annealed PVDF-TrFE 1 wt. % BNNT nanocomposite films for 5 days *in vitro*. Results are mean ± STD, * indicates a P-value of < 0.05, ** indicates a P-value < 0.01with respect to pristine non-poled samples.

Interestingly, it was observed that negatively charged annealed PVDF-TrFE films reduced tenocyte adhesion and proliferation relative to unpoled and positively charged pristine annealed PVDF-TrFE films (Figure 11). This can be explained as an electrostatic response. Since the membrane of tendon cells is also charged negatively it can be inferred that electrostatic repulsion can compete with integrin mediated cell attachment. In a similar study performed by Ribero et al. changes in cell proliferation were also observed as a consequence of the state of polarization of a PVDF surfaces (Ribeiro et al., 2018). Conversely, the anti-proliferative effect of negatively charged pristine annealed PVDF-TrFE films was not observed in negatively charged annealed PVDF-TrFE 1 wt. % BNNT nanocomposite films. In addition, it was clearly observed that cells attached and proliferate better on composite samples regardless of the polarization state. Again, it can be inferred that this effect is attributed to the modified substrate topography as a result of the BNNT incorporation and also to the electrically neutral surface presented by BNNTs that might act as an anchoring site for cells adhesion.

## Conclusion

Here, for the first time, evidence has been presented on increased β-phase formation and total crystallinity in PVDF-TrFE by incorporating BNNT as a second phase using modulated differential scanning calorimetry (MDSC). It also concludes that the annealing temperature appears to be critical for maximizing the β-phase formation. Incorporation of BNNT resulted in increased mechanical properties, melting and crystallization temperatures, and crystallinity via restriction of molecular movement. A poly acrylic acid/fibronectin functionalization approach was employed to promote cell adhesion and PVDF-TRFE/BNNT nanocomposites were shown to induce increased cell attachment and sustained proliferation relative to pristine PVDF-TrFE. Furthermore, the inclusion of BNNTs were observed to negate the anti-adhesive effects of negatively charged PVDF-TrFE surfaces. This study shows that PVDF-TrFE/BNNT nanocomposite hold great potential for tissue engineering applications.

## Author Contributions

AP and MAF were involved in doing experimental works and writing a paper. SAMT and MJPB were involved in direct supervision of the work.

### Conflict of Interest Statement

The authors declare that the research was conducted in the absence of any commercial or financial relationships that could be construed as a potential conflict of interest.
